# Nomogram to predict cause-specific mortality of patients with rectal adenocarcinoma undergoing surgery: a competing risk analysis

**DOI:** 10.1186/s12876-022-02131-1

**Published:** 2022-02-10

**Authors:** Xu Zhang, Fengshuo Xu, Yadi Bin, Tianjie Liu, Zhichao Li, Dan Guo, Yarui Li, Qiao Huang, Jun Lyu, Shuixiang He

**Affiliations:** 1 Department of Gastroenterology, The First Affiliated Hospital of Xi’an Jiaotong University, Xi’an, Shaanxi China; 2 Department of Clinical Research, The First Affiliated Hospital of Jinan University, Guangzhou, 510630 Guangdong Province China; 3 School of Public Health, Xi’an Jiaotong University Health Science Center, Xi’an, 710061 Shaanxi Province China; 4 Department of Urology, The First Affiliated Hospital of Xi’an Jiaotong University, Xi’an, Shaanxi China; 5 Center for Evidence-Based and Translational Medicine, Zhongnan Hospital of Wuhan University, Wuhan, China

**Keywords:** SEER, Rectal adenocarcinoma, Competing risk, Nomogram

## Abstract

**Background:**

Rectal adenocarcinoma is one of major public health problems, severely threatening people’s health and life. Cox proportional hazard models have been applied in previous studies widely to analyze survival data. However, such models ignore competing risks and treat them as censored, resulting in excessive statistical errors. Therefore, a competing-risk model was applied with the aim of decreasing risk of bias and thereby obtaining more-accurate results and establishing a competing-risk nomogram for better guiding clinical practice.

**Methods:**

A total of 22,879 rectal adenocarcinoma cases who underwent primary-site surgical resection were collected from the SEER (Surveillance, Epidemiology, and End Results) database. Death due to rectal adenocarcinoma (DRA) and death due to other causes (DOC) were two competing endpoint events in the competing-risk regression analysis. The cumulative incidence function for DRA and DOC at each time point was calculated. Gray’s test was applied in the univariate analysis and Gray’s proportional subdistribution hazard model was adopted in the multivariable analysis to recognize significant differences among groups and obtain significant factors that could affect patients’ prognosis. Next, A competing-risk nomogram was established predicting the cause-specific outcome of rectal adenocarcinoma cases. Finally, we plotted calibration curve and calculated concordance indexes (c-index) to evaluate the model performance.

**Results:**

22,879 patients were included finally. The results showed that age, race, marital status, chemotherapy, AJCC stage, tumor size, and number of metastasis lymph nodes were significant prognostic factors for postoperative rectal adenocarcinoma patients. We further successfully constructed a competing-risk nomogram to predict the 1-year, 3-year, and 5-year cause-specific mortality of rectal adenocarcinoma patients. The calibration curve and C-index indicated that the competing-risk nomogram model had satisfactory prognostic ability.

**Conclusion:**

Competing-risk analysis could help us obtain more-accurate results for rectal adenocarcinoma patients who had undergone surgery, which could definitely help clinicians obtain accurate prediction of the prognosis of patients and make better clinical decisions.

## Background

Cancer is a one of the biggest problems facing public health around the world, with one in four deaths in the USA being due to cancer [1]. Colorectal carcinoma is the second most common malignancy in females, with an annual incidence of 15 cases per 100,000, and the third most common malignancy in males worldwide, with an annual incidence of 22 cases per 100,000 [2]. About one-third of colorectal cancers are found in the rectum area [3]. Cancerous lesions found within 12 cm from the anal verge when using a proctoscope are defined as rectal cancer, and most of rectal cancer are histologically characterized by adenocarcinoma [4]. Moreover, the risk of local recurrence is higher in rectal adenocarcinoma than in colon adenocarcinoma [5]. The incidence rate of rectal adenocarcinoma was historically low in China, but changes in lifestyle and nutritional habits in recent years have resulted in the rate increasing. Rectal adenocarcinoma has been a fairly common malignant tumor in the USA, which is diagnosed in nearly 50,000 patients annually [6]. The nomogram is regarded as an effective analytical and statistical tool to predict the outcomes of patients accurately. Because rectal adenocarcinoma undergoing surgical resection varies largely in prognosis, in this research, we sought to construct nomograms for predicting survival outcomes in postoperative patients with rectal adenocarcinoma.

A person is usually exposed to many causes of failure, but exactly one event contributed to the final failure. This is called competing risk. In this situation, the occurrence of any other event can be hindered by one type of event [7]. Nevertheless, Cox regression analysis, which is commonly applied for survival analysis, ignores competing risks and treats them as censored. When the widely used survival analysis methods are applied, it might contribute to inaccurate and biased results [8, 9]. The Fine and Gray’s proportional subdistribution hazard model could be used to test a covariate when competing risks are presented, which guarantees that the conclusions we draw are unbiased and could be precisely interpreted [10, 11].

This research investigated death due to rectal adenocarcinoma (DRA) as the event of interest and death due to other causes (DOC) as a competing event. A competing-risk model was used to analyze the prognosis and establish a nomogram model of rectal adenocarcinoma undergoing surgery with the aim of obtaining more-accurate results.

## Methods

### SEER database

The data referenced were published on the Surveillance, Epidemiology and End Results (SEER) database. It collects data about morbidity, prevalence, and survival of cancer from population-based cancer registries covering about 30% of the US population [12]. The SEER database is a preferential source of cancer surveillance data as well as analytical tools, and is an expert in collecting, analyzing, illuminating, and announcing dependable population-based statistics [13, 14]. The database we applied was the Incidence—SEER 18 Regs Custom Data (with additional treatment fields), Nov 2018 Sub (1975–2016 varying). We were permitted to access the data using the ID number 15277-Nov2019 by way of Internet access direction.

### Cases selection

**Fig. 1:**
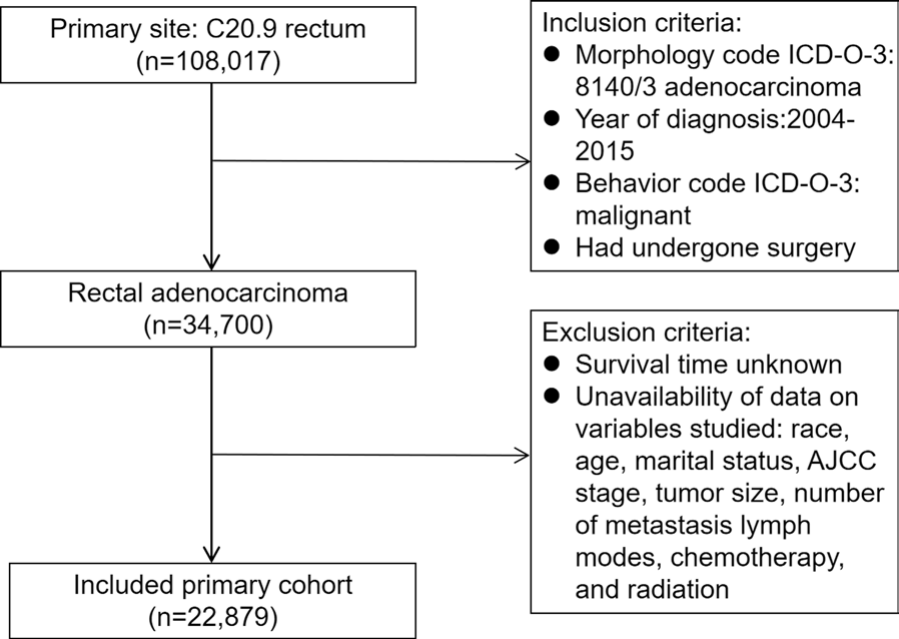
Patient inclusion and exclusion process of in the SEER database

Applying the criteria in ICD-O-3 revealed 108,017 patients with rectal cancer in the SEER database. The inclusion criteria for this research were: (1) adenocarcinoma, (2) diagnosed between 2004 and 2015, (3) had undergone surgery, and (4) presence of malignant behavior. The exclusion criteria for this study were (1) unavailability of data on race, age, sex, marital status, AJCC stage, tumor size, number of metastatic lymph nodes, radiation, and chemotherapy and (2) unknown survival time. Applying these criteria resulted in 22,879 patients being enrolled in this study (Fig. 1).

### Variables

Information on the following ten variables was obtained from the SEER database: race, sex, age, marital status, AJCC stage, tumor size, number of metastatic lymph nodes, radiation, and chemotherapy. Race was categorized into black, white, and others. AJCC stage was classified into level I to level IV. Similarly, tumor size was categorized into < 4 cm, 4–8 cm, and ≥ 8 cm, and number of metastatic lymph nodes was classified into 0, 1–3, 4–8, and > 8. The outcomes of patients were categorized into the following three situations: alive, DRA, and DOC.

### Statistical analysis and construction of a competing-risk nomogram

The eligible cases were categorized into training set (n = 16,015) and validation set (n = 6,864) randomly. In the competing-risk model, cause-specific death and other causes of death were two competing endpoint events. The cumulative incidence function (CIF) for death due to rectal adenocarcinoma (DRA) and death due to other causes (DOC) at each time point was calculated according to above-mentioned nine variables. Meanwhile, CIF curves for every variable were plotted using SAS software, which presented the cumulative incidence of various outcomes in DRA and DOC patients over time since diagnosis. Gray’s test was applied in the univariate analysis and Gray’s proportional subdistribution hazard model was adopted in the multivariable analysis to recognize significant differences among groups and obtain significant factors that could affect patients’ prognosis. Next, a competing-risk nomogram was established to predict the DRA probability [15]. Finally, we plotted calibration curve and calculated concordance indexes (c-index) to evaluate performance of the nomogram model [16, 17]. If the calibration curve was close to a 45-degree straight line, the nomogram model was proved to have excellent predicting performance [18]. The analyses were performed using SAS and R statistical software version 3.6.2 (https://www.r-project.org) statistical software. *P* < 0.05 was regarded as indicative of statistically significant effects.

## Results

### Patient characteristics

22,879 patients were included finally, of whom 5,735 (25.07%) died due to rectal adenocarcinoma and 2529 (11.05%) were DOC patients. 9345 (40.85%) of all patients were older than 65 years old. In the total patients, 13,766 (60.17%) were male and 18,627 (81.42%) were white. The proportion of AJCC stage was 19.74%, 28.48%, 41.94%, and 9.84% for stage I, stage II, stage III, and stage IV, respectively. Most patients’tumor size stayed at 4–8 cm (47.80%), followed by < 4 cm (44.08%) and ≥ 8 cm (8.12%). Besides, there were more patients who did not have lymph node metastasis (62.06%). A total of 15,322 patients (66.97%) had received radiation, and 16,632 patients (72.70%) were treated with chemotherapy.

The proportions of patients who were older than 65 years old was 40.87% (n = 2344) and 38.16% (n = 965) in the DRA group and DOC group, respectively. The DRA cohort comprised 3506 males and 2229 females, while there were 1556 males and 973 females in the DOC group. The proportions of married status were 55.26% and 53.30% in the DRA group and DOC patients, respectively. Most RAs were 4–8 cm in both groups, accounting for 52.31% and 50.73%, respectively. The proportions of patients who received radiation was 62.93% (n = 3609) and 50.22% (n = 1270) in the DRA cohort and DOC group, respectively. Chemotherapy was applied to 72.70% (n = 16,632) of the total cohort and 74.40% (n = 4267) of the DRA group (Table 1).

**Table 1:** Characteristics and demographics of patients with rectal adenocarcinoma undergoing surgery

Variables	Classification	Total (%)	Cause-specific death (%)	Death due to other causes (%)
n		22,879	5735 (25.07)	2529 (11.05)
Age (mean ± SD)		61.46 ± 12.988	63.33 ± 13.70	71.55 ± 11.60
Age				
	< 65	13,534 (59.15)	3391 (59.13)	1564 (61.84)
	≥ 65	9345 (40.85)	2344 (40.87)	965 (38.16)
Race				
	White	18,627 (81.42)	4587 (79.98)	2117 (83.71)
	Black	1768 (7.73)	554 (9.66)	196 (7.75)
	Other	2484 (10.86)	594 (10.36)	216 (8.54)
Sex				
	Male	13,766 (60.17)	3506 (61.13)	1556 (61.53)
	Female	9113 (39.83)	2229 (38.87)	973 (38.47)
MS				
	Married	14,077 (61.53)	3169 (55.26)	1348 (53.30)
	Unmarried	8802 (38.47)	2566 (44.74)	1181 (46.70)
AJCC				
	I	4517 (19.74)	472 (8.23)	700 (27.68)
	II	6515 (28.48)	1165 (20.31)	855 (33.81)
	III	9595 (41.94)	2611 (45.53)	847 (33.49)
	IV	2252 (9.84)	1487 (25.93)	127 (5.02)
TS				
	< 4 cm	10,086 (44.08)	2155 (37.58)	1083 (42.82)
	4–8 cm	10,936 (47.80)	3000 (52.31)	1283 (50.73)
	≥ 8 cm	1857 (8.12)	580 (10.11)	163 (6.45)
LN				
	0	14,198 (62.06)	2253 (39.29)	1747 (69.08)
	1–3	5330 (23.30)	1752 (30.55)	503 (19.89)
	4–8	2375 (10.38)	1117 (19.48)	218 (8.62)
	> 8	976 (4.27)	613 (10.69)	61 (2.41)
Radiation				
	None	7557 (33.03)	2126 (37.07)	1259 (49.78)
	Yes	15,322 (66.97)	3609 (62.93)	1270 (50.22)
Chemotherapy				
	None	6247 (27.30)	1468 (25.60)	1243 (49.15)
	Yes	16,632 (72.70)	4267 (74.40)	1286 (50.85)

MS, marital status; TS, tumor size; LN, number of metastasis lymph nodes

### Univariate analysis

**Fig. 2:**
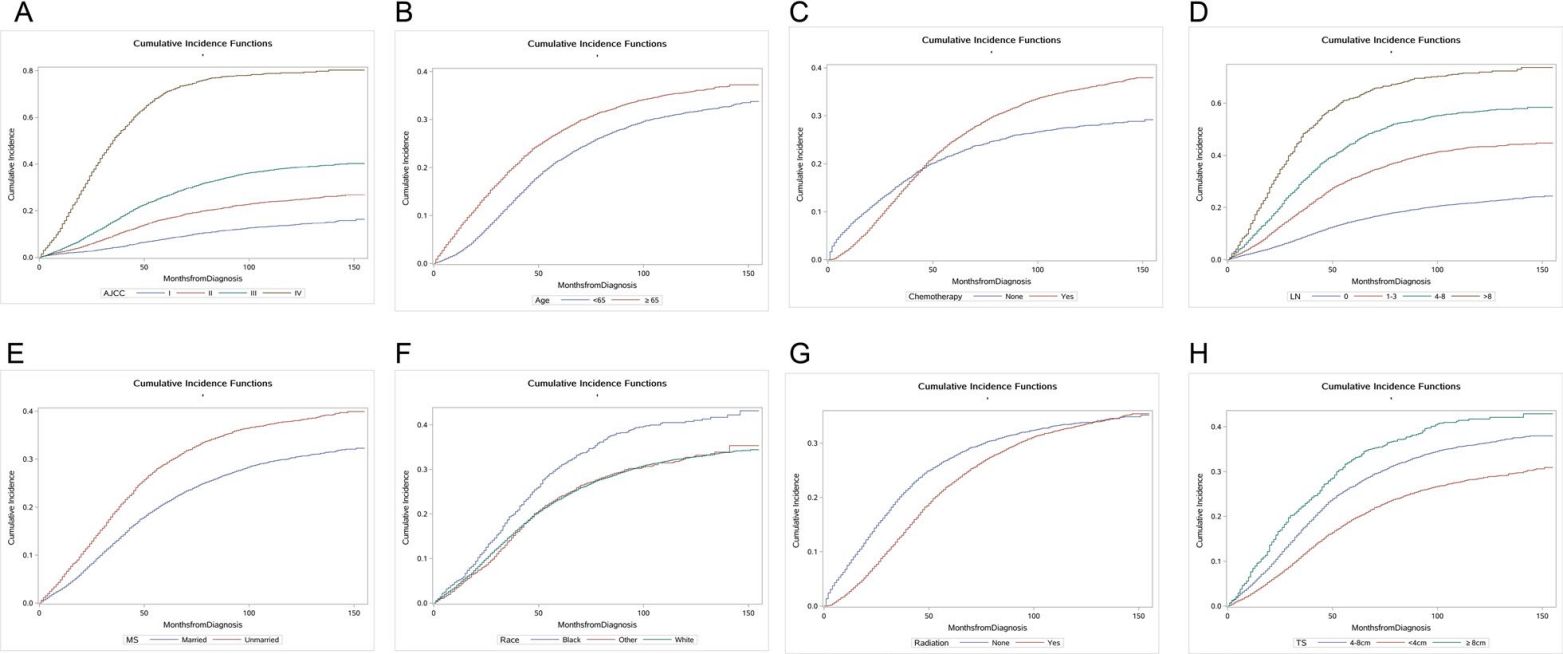
Cumulative incidence functions of cause-specific death according to AJCC stage (**A**), age (**B**),  chemotherapy (**C**), number of metastasis lymph nodes (**D**), marital status (**E**), race (**F**), radiation (**G**), and tumor size (**H**)

**Fig. 3:**
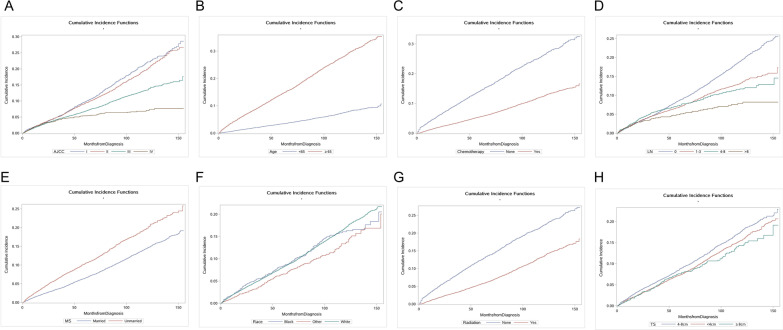
Cumulative incidence functions of other causes of death according to AJCC stage (**A**), age (**B**),  chemotherapy (**C**), number of metastasis lymph nodes (**D**), marital status (**E**), race (**F**), radiation (**G**), and tumor size (**H**)

The univariate analysis showed that race, marital status, age, tumor size, number of metastatic lymph nodes, AJCC stage, chemotherapy, and radiation were significant prognostic factors in rectal adenocarcinoma patients undergoing surgery. Meanwhile, we calculated the 1-year, 3-year, and 5-year cumulative incidence of DRA and DOC, as presented in Table 2. The CIF curves of DRA are presented in Fig. 2A–H, while that of DOC are shown in Fig. 3A–H.

**Table 2:** Univariate analysis of prognostic factors in patients with rectal adenocarcinoma undergoing surgery

Variables	Classification	Cause-specific death (%)				Death due to other causes (%)			
		1-year (95% CI)	3-year (95% CI)	5-year (95% CI)	*P* value	1-year (95% CI)	3-year (95% CI)	5-year (95% CI)	*P* value
Age					< 0.001				< 0.001
	< 65	2.39 (2.34–2.45)	12.21 (11.95–12.48)	21.55 (21.08–22.02)		0.67 (0.65–0.68)	2.04 (1.99–2.09)	3.39 (3.30–3.47)	
	≥ 65	7.39 (7.17–7.60)	19.41 (18.88–19.94)	27.43 (26.70–28.16)		3.70 (3.58–3.81)	9.04 (8.76–9.31)	14.39 (13.93–14.85)	
Race					< 0.001				0.0027
	White	4.44 (4.37–4.50)	14.92 (14.70–15.14)	23.34 (23.00–23.69)		1.99 (1.96–2.02)	5.04 (4.96–5.12)	8.14 (8.00–8.29)	
	Black	5.23 (4.39–6.06)	19.36 (16.48–22.25)	30.72 (26.31–35.12)		1.99 (1.66–2.31)	5.53 (4.60–6.47)	8.38 (6.85–9.90)	
	Other	3.86 (3.41–4.31)	14.10 (12.49–15.71)	23.89 (21.16–26.63)		1.22 (1.07–1.36)	3.63 (3.17–4.09)	6.58 (5.67–7.50)	
Sex					0.1107				0.1401
	Male	4.42 (4.32–4.51)	15.05 (14.75–15.35)	24.28 (23.79–24.76)		1.96 (1.91–2.00)	4.90 (4.79–5.01)	8.19 (7.99–8.38)	
	Female	4.46 (4.32–4.60)	15.36 (14.90–15.83)	23.51 (22.80–24.22)		1.83 (1.77–1.89)	4.97 (4.80–5.13)	7.70 (7.43–7.98)	
MS					< 0.001				< 0.001
	Married	3.40 (3.34–3.47)	12.76 (12.51–13.02)	20.87 (20.45–21.29)		1.50 (1.47–1.53)	3.80 (3.72–3.88)	6.51 (6.36–6.67)	
	Unmarried	6.08 (5.89–6.28)	19.06 (18.49–19.63)	29.00 (28.15–29.85)		2.56 (2.47–2.64)	6.74 (6.51–6.97)	10.40 (10.02–10.78)	
AJCC					< 0.001				< 0.001
	I	1.76 (1.64–1.87)	4.23 (3.94–4.51)	7.93 (7.36–8.49)		2.00 (1.87–2.13)	5.52 (5.16–5.89)	9.77 (9.09–10.45)	
	II	2.67 (2.55–2.79)	9.52 (9.09–9.95)	16.42 (15.66–17.19)		1.99 (1.90–2.08)	5.28 (5.04–5.53)	9.30 (8.83–9.76)	
	III	4.21 (4.08–4.34)	15.74 (15.29–16.20)	26.14 (25.38–26.89)		1.77 (1.71–1.82)	4.56 (4.42–4.71)	6.80 (6.56–7.04)	
	IV	15.82 (14.06–17.58)	51.47 (47.83–55.11)	70.71 (67.22–74.19)		2.05 (1.78–2.31)	4.21 (3.65–4.77)	5.38 (4.62–6.14)	
TS					< 0.001				0.0007
	< 4 cm	2.93 (2.85–3.02)	11.48 (11.15–11.80)	19.47 (18.91–20.02)		1.72 (1.67–1.77)	4.46 (4.33–4.60)	7.28 (7.04–7.51)	
	4–8 cm	5.15 (5.01–5.28)	17.41 (16.98–17.84)	26.74 (26.09–27.39)		2.16 (2.10–2.22)	5.44 (5.29–5.59)	8.74 (8.48–9.01)	
	≥ 8 cm	8.40 (7.16–9.64)	22.38 (19.31–25.46)	32.87 (28.38–37.37)		1.41 (1.18–1.63)	4.44 (3.68–5.19)	7.61 (6.18–9.04)	
LN					< 0.001				< 0.001
	0	2.59 (2.54–2.65)	8.65 (8.47–8.84)	14.74 (14.41–15.06)		1.83 (1.79–1.87)	4.95 (4.84–5.05)	8.73 (8.52–8.94)	
	1–3	5.19 (4.91–5.46)	19.25 (18.30–20.19)	31.39 (29.92–32.85)		1.98 (1.87–2.09)	4.76 (4.49–5.03)	6.93 (6.51–7.35)	
	4–8	9.33 (8.27–10.39)	30.34 (27.51–33.17)	44.68 (41.06–48.30)		2.28 (2.00–2.56)	5.59 (4.90–6.28)	7.40 (6.45–8.35)	
	> 8	15.10 (11.19–19.01)	48.35 (40.20–56.51)	62.11 (53.82–70.40)		1.75 (1.22–2.27)	3.93 (2.74–5.12)	4.99 (3.41–6.57)	
Radiation					< 0.001				< 0.001
	None	7.95 (7.66–8.24)	20.13 (19.45–20.80)	27.27 (26.37–28.16)		3.47 (3.34–3.61)	8.20 (7.89–8.51)	12.51 (12.02–13.01)	
	Yes	2.70 (2.65–2.76)	12.71 (12.47–12.95)	22.36 (21.94–22.79)		1.14 (1.11–1.16)	3.29 (3.23–3.36)	5.69 (5.56–5.82)	
Chemotherapy					< 0.001				< 0.001
	None	7.91 (7.56–8.26)	16.34 (15.66–17.03)	22.00 (21.08–22.91)		3.91 (3.73–4.09)	9.25 (8.83–9.67)	14.26 (13.59–14.92)	
	Yes	3.13 (3.08–3.19)	14.76 (14.51–15.01)	24.84 (24.42–25.26)		1.15 (1.13–1.17)	3.27 (3.21–3.33)	5.53 (5.41–5.64)	

MS, marital status; TS, tumor size; LN, number of metastasis lymph nodes

### Multivariable analysis

In the multivariable analysis, the Fine and Gray’s proportional subdistribution hazard model was used to obtain significant prognostic factors. The results indicated that age, race, marital status, AJCC stage, tumor size, number of metastatic lymph nodes, and chemotherapy were significant prognostic factors affecting survival. Compared with married patients, patients who were at unmarried status had higher risk of DRA, with subdistribution hazard ratios (sdHRs) of 1.299 (95% CI 1.23–1.372). Compared with stage I, patients who stayed at advanced AJCC stages had higher rates of cause-specific mortality, with sdHRs of 2.008 (95% CI 1.792–2.25), 2.086 (95% CI 1.822–2.388), and 7.791 (95% CI 6.762–8.976), for stage II, III, and IV, respectively. Patients who had larger tumor had worse prognosis, as well as more metastatic lymph nodes. Patients who underwent chemotherapy, had lower risk of cancer-specific mortality, with sdHRs of 0.853 (95% CI 0.78–0.933). For the DOC group, The results can be understood in the same way (Table 3).

**Table 3:** Proportional subdistribution hazards models for rectal adenocarcinoma undergoing surgery

Variables	Classification	Cause-specific death				Death due to other causes			
		Coefficient	HR	95% CI	*P* value	Coefficient	HR	95% CI	*P* value
Age									
	< 65	Reference				Reference			
	≥ 65	0.383	1.467	1.388–1.550	< 0.001	1.315	3.723	3.402–4.075	< 0.001
Race									
	Black	Reference				Reference			
	White	− 0.208	0.812	0.739–0.893	< 0.001	− 0.072	0.931	0.802–1.080	0.346
	Other	− 0.249	0.779	0.690–0.880	< 0.001	− 0.264	0.768	0.632–0.933	0.008
MS									
	Married	Reference				Reference			
	Unmarried	0.277	1.319	1.249–1.392	< 0.001	0.248	1.281	1.184–1.387	< 0.001
AJCC									
	I	Reference				Reference			
	II	0.723	2.060	1.838–2.309	< 0.001	0.107	1.113	1.000–1.239	0.050
	III	0.748	2.113	1.845–2.420	< 0.001	− 0.083	0.920	0.771–1.099	0.357
	IV	2.044	7.720	6.700–8.895	< 0.001	− 0.585	0.557	0.438–0.708	< 0.001
TS									
	< 4 cm	Reference				Reference			
	4–8 cm	0.068	1.071	1.011–1.134	0.020	0.160	1.173	1.080–1.274	< 0.001
	≥ 8 cm	0.312	1.366	1.240–1.505	< 0.001	0.104	1.110	0.939–1.313	0.223
LN									
	0	Reference				Reference			
	1–3	0.542	1.719	1.564–1.889	< 0.001	− 0.023	0.977	0.825–1.158	0.791
	4–8	0.886	2.426	2.190–2.686	< 0.001	− 0.020	0.980	0.804–1.194	0.841
	> 8	1.196	3.305	2.927–3.733	< 0.001	− 0.355	0.701	0.522–0.942	0.019
Radiation									
	None	Reference				Reference			
	Yes	− 0.035	0.965	0.895–1.041	0.357	− 0.053	0.948	0.831–1.081	0.425
Chemotherapy									
	None	Reference				Reference			
	Yes	− 0.211	0.810	0.741–0.885	< 0.001	− 0.534	0.586	0.514–0.669	< 0.001

MS, marital status; TS, tumor size; LN, number of metastasis lymph nodes

### Construction and validation of the nomogram

**Fig. 4:**
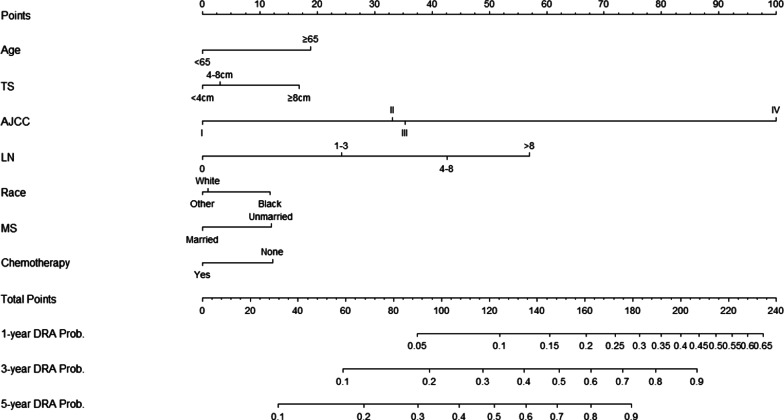
Nomogram predicting 1-, 3-, and 5-year cause-specific death of patients with rectal adenocarcinoma undergoing surgery

**Fig. 5:**
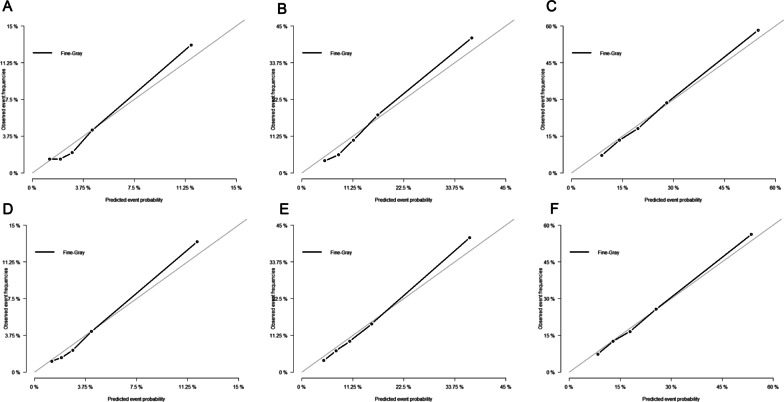
Calibration plot of the nomogram for 1-, 3- and 5-year mortality prediction of the training set (**A**–**C**) and validation set (**D**–**F**). Abbreviations: MS, marital status; TS, tumor size; LN, number of metastasis lymph nodes

A competing-risk nomogram was constructed to predict the 1-year, 3-year and 5-year cause-specific death probabilities, as shown in Fig. 4. To use the nomogram, draw a vertical lines between the variable's rows and the top “points” line to locate the values of variables on the variable rows and then draw vertical lines straight in order to gain the points of these variables. Then, add up each variable’s point and the total point could be calculated. Draw a vertical line between the “Total Points” line and the “1-year DRA Prob.”, “3-year DRA Prob.”, or “5-year DRA Prob.” line. In the end, the 1-year, 3-year and 5-year cause-specific death probabilities were calculated. The calibration curve for the nomogram of the training cohort and the validation cohort was shown in Fig. 5A–F. The calibration curve was close to a 45-degree straight line, which indicates the competing-risk nomogram model was calibrated properly. The C-indexes at 1-year, 3-year, and 5-year in the training set were 0.782, 0.768, and 0.742, respectively. For the test cohort, the 1-year, 3-year, and 5-year C-indexes were 0.769, 0.769, and 0.745.

## Discussion

In this study, DOC occurred in 2529 of 8264 patients, accounting for about one-third of deaths. Cox proportional-hazards models, which have been widely applied in previous studies, treat these competing events as censored data, thereby contributing to the risk of bias. In contrast, the competing-risk model utilized in the present study greatly decreased the bias, and so more-accurate results were obtained for rectal adenocarcinoma that will allow better guidance of clinical practice. A parameter termed as ‘cumulative incidence’, calculated by the competing-risk model, has been recommended to conquer the defects of traditional survival analysis for over 20 years [19–24]. The Fine and Gray’s model could be used to test a covariate when competing risks are presented [10]. Using appropriate techniques for the competing-risk analysis guarantees that the conclusions are unbiased and could be correctly interpreted [11]. We used a competing-risk model to obtain more-accurate information about rectal adenocarcinoma by decreasing the risk of bias, so our research has a profound impact on clinical practice.

Many previous papers investigated the prognostic impact of race, age, sex and marital status. For race, some studies [25–28] have indicated that overall survival is better among whites than blacks. However, another study, which applied Cox regression analysis to data from the SEER database [29], found that race was not a significant prognostic factor for colorectal adenocarcinoma. In the current research, both the univariate and multivariable analyses showed that race could significantly affect survival outcomes of patients with rectal adenocarcinoma who had undergone surgery. There are gender differences in cancer susceptibility, which is most-consistent but least-understood in cancer research [30, 31]. The current study found that sex could not statistically affect postoperative patients’ prognosis in the competing-risk analysis. Previous studies [27, 29, 32, 33] using Cox regression analysis have suggested that being older is a significant risk factor for survival. The univariate and multivariable analysis in the current study all demonstrated that age was a statistically significant factor for the prognosis of this disease; this consistency across the different methods indicates the reliability of this finding. One study indicated that marriage was related with better prognosis of rectal cancer patients undergoing surgery, but unmarried patients, especially widowed patients, had higher risk of cancer-specific mortality [34]. Our research also presented an increased risk of cause-specific mortality associated with the unmarried status. We hypothesize that unmarried status affects patients’ prognosis indirectly by decreasing mental health and well-being [34–36].

According to previous studies, tumor size and metastatic lymph nodes seemed to be risk factors that strongly affect survival in rectal adenocarcinoma. One study [37] indicated that tumor size significantly affect overall survival of patients with colorectal adenocarcinoma. Another study [38] identified a tumor size of < 5 cm as a strong prognostic factor for rectal adenocarcinoma. However, the very small samples and the use of Cox analysis contributed to excessive statistical errors in those studies. Studies [27, 33] have also found that the HRs for overall survival in rectal cancer decreased with an increasing number of lymph nodes examined. The univariate and multivariable analyses in the current study showed that a larger tumor, and more metastatic lymph nodes were risk factors that strongly affect overall survival in rectal adenocarcinoma, which agrees with the previous findings. However, the much larger sample and the application of multiple analytical methods make the results reported in this paper much more reliable.

For locally advanced rectal adenocarcinoma patients, radiochemotherapy is considered as a reliable and practical treatment [39]. On the basis of the most-recent National Comprehensive Cancer Network guidelines for colon cancer published in 2012, neoadjuvant chemoradiotherapy followed by total mesorectal excision is the current standard therapy for advanced low- and mid-rectal adenocarcinoma [40]. The reported remission rate has been as high as 48% [41]. Neoadjuvant concurrent chemoradiation has contributed not only to higher resection rates with minimal side effects but also to decreasing recurrence rate and tumor size [37]. The present study found that chemotherapy could improve rectal adenocarcinoma patients’ outcomes. However, there is no guarantee that patients’ characteristics were properly recorded in the SEER database, and the chemotherapy effect may result from those who do not receive chemotherapy being not suitable for chemotherapy, rather than a real effect of chemotherapy. Radiotherapy has played a crucial role in the management and local control of rectal adenocarcinoma for several decades [42]. However, some studies [26, 43] have found no difference in survival between surgery alone and surgery combined with radiotherapy. In the present study, the multivariable analysis revealed that radiation did not improve prognosis.

In this paper, the nomogram we established was proved to be an practical and feasible tool for evaluating specific events probabilities and clinical decision-making via a user-friendly graph with easily available clinicopathological data. Besides, the established nomogram showed good validation, but we need to further validate the efficiency of this nomogram based on large-scale cohorts in the future.

Of course, this study has some limitations. One of them was that some important characteristics of patients are not included in the SEER database, and we did not select all potential prognostic factors in the database in this study. Besides, SEER lacks detailed information on some aspects, such as type and dose of radiotherapy and chemotherapy, which could definitely decrease the reliability of our conclusion [44]. Chemotherapy and radiation are not well collected by SEER, so there is substantial misclassification (https://seer.cancer.gov/data-software/documentation/seerstat/nov2020/treatment-limitations-nov2020.html). And some very important prognostic factors, such as more detailed location, the grade of differentiation degree and CEA status were not included in the SEER database, which could decrease clinical practicability. Especially, although many studies have found race was an important prognostic factor for survival outcomes, it is possible that the results were subject to confounding from differences in income, socio-economic status, insurance status, genetic biomarkers, and comorbidities, etc. [45]. Some studies attempted to limit the extent of confounding as far as possible, whereas we were unable to identify and control those potential confounders beforehand due to the restrictions of the SEER database. Thus, the generalizability of the nomogram still needs to be validated by further studies. More importantly, nomograms should be interpreted and applied with caution in clinical practice. On the other hand, in future studies, we would optimize the selection of clinically and statistically significant predictors and promote the clinical practicability of the nomogram.

## Conclusion

Competing-risk analysis greatly decreases the risk of bias associated with common analytical methods. This study used competing-risk analysis to obtain more-accurate results for rectal adenocarcinoma patients undergoing surgery. The results showed that age, marriage, race, marital status, AJCC stage, tumor size, number of metastasis lymph nodes, and chemotherapy were significant prognostic factors for these patients. We further successfully constructed a competing-risk nomogram to predict the cause-specific survival of rectal adenocarcinoma patients. The validation also demonstrated the accuracy of the model, which could definitely help clinicians obtain accurate prediction of the prognosis of patients and make better clinical decisions.

## Data Availability

The data of this study are available from SEER database. We were permitted to access the data using the ID number 15277-Nov2019 by way of Internet access direction, so they are not publicly available. However, data are available from the authors with permission of SEER database.

## Abbreviations

SEER: Surveillance, Epidemiology, and End Results
DRA: Death due to rectal adenocarcinoma
DOC: Death due to other causes
CIF: Cumulative incidence function
c-index: Concordance indexes
sdHRs: Subdistribution hazard ratios
